# Delayed ITU discharge: causes and impact

**DOI:** 10.1186/cc9884

**Published:** 2011-03-11

**Authors:** M Flower, A Kaliappan

**Affiliations:** 1 West Suffolk Hospital, Bury St Edmunds, UK

## Introduction

ITU resources represent 13% of hospital costs. Patients requiring ITU should be admitted promptly. However, those identified as suitable for discharge to the ward should also be transferred swiftly.

## Methods

A retrospective study of notes for 269 people admitted to the ITU between April and September 2010. Variables included length of ITU stay, discharge destination, reason for admission to ITU, primary pathology, disease severity on admission (APACHE II), health on discharge (MEWS) and ward bed availability.

## Results

Most discharges occur out of hours (64%). The average length of ITU stay is 90 hours and the average discharge delay is 26 hours. As length of stay increases, so too does discharge delay. Discharge delay was not significantly correlated with increased hospital mortality. Those discharged to the ward were delayed by an average of 32 hours. Primary reasons for ITU admission included monitoring, diagnosis and support of physiological function, with the latter by far the commonest. Discharge delay was significantly longer for those admitted in order to establish a diagnosis (40 hours). Discharge delay was very short for biliary and cerebral disease, at 3 and 2 hours respectively, but much longer for pneumonia, acute renal failure and heart failure, at 38, 58 and 72 hours. No correlation was found between discharge delay and APACHE II score on admission or MEWS score on discharge.

## Conclusions

ITU patients have complex care needs and transition through several departments. We focused on ITU factors and found discharge was delayed by long ITU stay, acute renal failure, heart failure, pneumonia and a lack of diagnosis on admission. The commonest ward factors are bed availability, emergency department activity, ward discharge practices and patient deterioration. In the community there are finite resources for special care. ITU patients should be prioritised for ward beds. Multispeciality involvement on intensive care and the presence of advanced diagnostic facilities on site, such as CT and angiography, would expedite diagnosis. Adequate step-down facilities, such as dialysis and respiratory support, should be available in order to accept patients with complex needs and would enable earlier and safer discharge from intensive care.

